# Unraveling Cherubism: Examining the Classical "Eye to Heaven" Phenomenon

**DOI:** 10.7759/cureus.62277

**Published:** 2024-06-12

**Authors:** Parmarth M Sonpal, Bhushan P Mundada, Nitin Bhola, Chetan Gupta, Ketan Dodal

**Affiliations:** 1 Oral and Maxillofacial Surgery, Sharad Pawar Dental College and Hospital, Datta Meghe Institute of Higher Education and Research, Wardha, IND

**Keywords:** orbit, mandible, osteoclast, fibro-osseous lesion, cherubism

## Abstract

Cherubism, an infrequent disorder with paramount autosomal importance, predominantly targets the mandible, with occasional involvement of the maxilla. Manifesting in childhood, it typically improves over time but never fully resolves in adulthood. Clinically, it presents as a uniform enlargement of the bones, and when the upper jaw is involved, it can create a cherub-like appearance with exposure to the sclera. As the volume grows, it can cause symptoms such as dental misalignment, delayed tooth eruption, speech difficulties, and tooth loss, in addition to psychological and cosmetic effects that require medical attention. The disorder progresses naturally in youngsters, exhibiting phases of expansion, stabilization, and regression. Cherubism initially is encountered in early childhood, reaches its peak during early years, balances out around puberty, and then steadily recedes after that. We describe the example of a male patient, age 20, who sought correction due to worries about his appearance. He had a bilateral mandibular angle and malar edema. The patient's aesthetic discontent was satisfactorily resolved with surgical intervention, and further pharmaceutical therapy was implemented during follow-up visits.

## Introduction

According to Jones and Pinborg et al., cherubism is a benign bone condition marked by the symmetrical, painless swelling of the jaw. Maxillary tuberosities or swelling in the mandibular body usually emerge between the ages of two and seven in children with this disorder, although they appear normal at birth. Men are affected by the illness twice as often as women [1,2]. Cherubism typically presents with a rounded face and deformities in the upper jaw, characterized by the swelling of the middle and lower sections of the facial skeleton. The floor of the orbit, the front part of the maxilla, and the suborbital bone can all be impacted by fibrous alterations. The palate becomes deformed and has a V-shaped shape as a result of the augmentation of the alveolar process. The disorder known as cherubism was coined by Jones because of the distinctive facial look caused by the upward displacement of the eyeballs [1]. According to the literature, cherubism is an autoinflammatory disease that only affects the craniofacial bones [3]. Three stages of development are commonly experienced by children: expansion, stabilization, and recession. Early childhood is when these developmental stages first appear, get stronger as children get older, and then level out at about age seven. After a time of relative balance throughout puberty, these mechanisms begin to diminish as a person enters adolescence and beyond [4]. Cherubism is caused due to mutations in the SH3BP2 gene, which is responsible for encoding of adaptor protein SH3BP2. There is an abundance of information in the literature regarding the manifestations of cherubism, ranging from showing no symptoms to asymptomatic typical cases to aggressive types showing orbital damage, or even, in extreme circumstances, resulting in death [5,6].

## Case presentation

**Figure 1:**
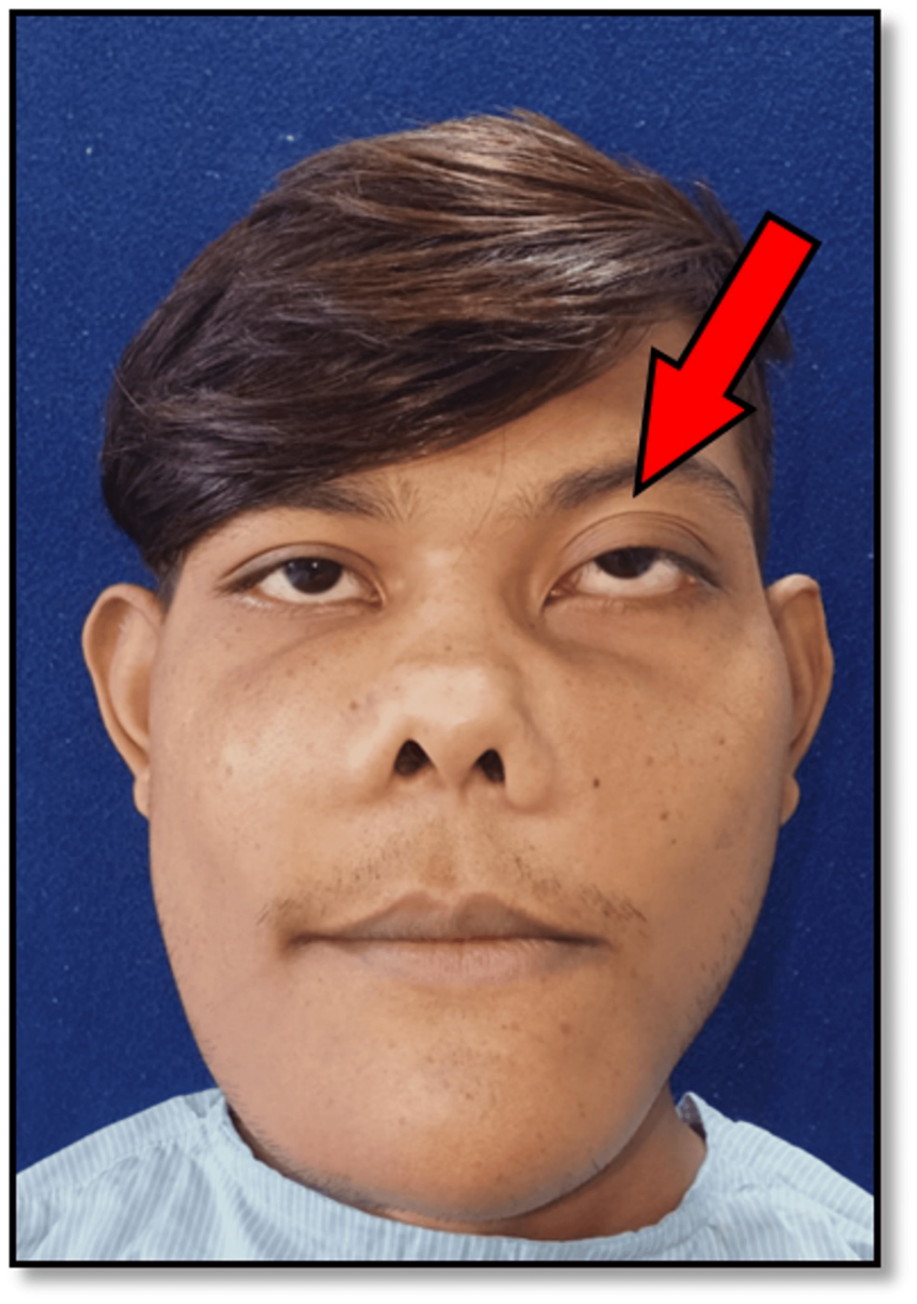
Frontal view showing the classical “Eye to Heaven” appearance

A 20-year-old male patient presented to us with a chief complaint of facial and jaw swelling persisting for approximately seven to eight years. Clinical examination revealed symmetrical swelling involving the bilateral mandibular body, angle area, symphysis, and malar region. The swelling exhibited a non-painful, bone-like consistency upon palpation. The patient experienced mild paresthesia on the ala of the nose over the right side and has had blurring of vision in the left eye for the past two to three years approximately. Evaluation of the temporomandibular joint (TMJ) indicated good function with adequate mouth opening. Intraoral examination revealed the absence of mandibular permanent incisors and grade II mobility of the left mandibular lateral incisor, with stable bilateral occlusion. Notably, the patient presented with the characteristic "Eye to Heaven" facial appearance (Figure 1).

In addition to routine blood investigations, we conducted supplementary tests which showed a modest increase in serum alkaline phosphatase, intact parathyroid hormone, and vitamin D as shown in Table 1.

**Table 1:** Blood investigations with their normal range IPTH: intact parathyroid hormone

Investigations	Observed value	Normal value
Serum calcium	10.3 mg/dL	8.5-10.5 mg/dL
Serum ionic calcium	4.88 mg/dL	4.5-5.6 mg/dL
IPTH	65.7 pg/mL	14-65 pg/mL
Alkaline phosphatase	159 IU/L	44-147 IU/L
Vitamin D	45.6 ng/mL	20-40 ng/mL

**Figure 2:**
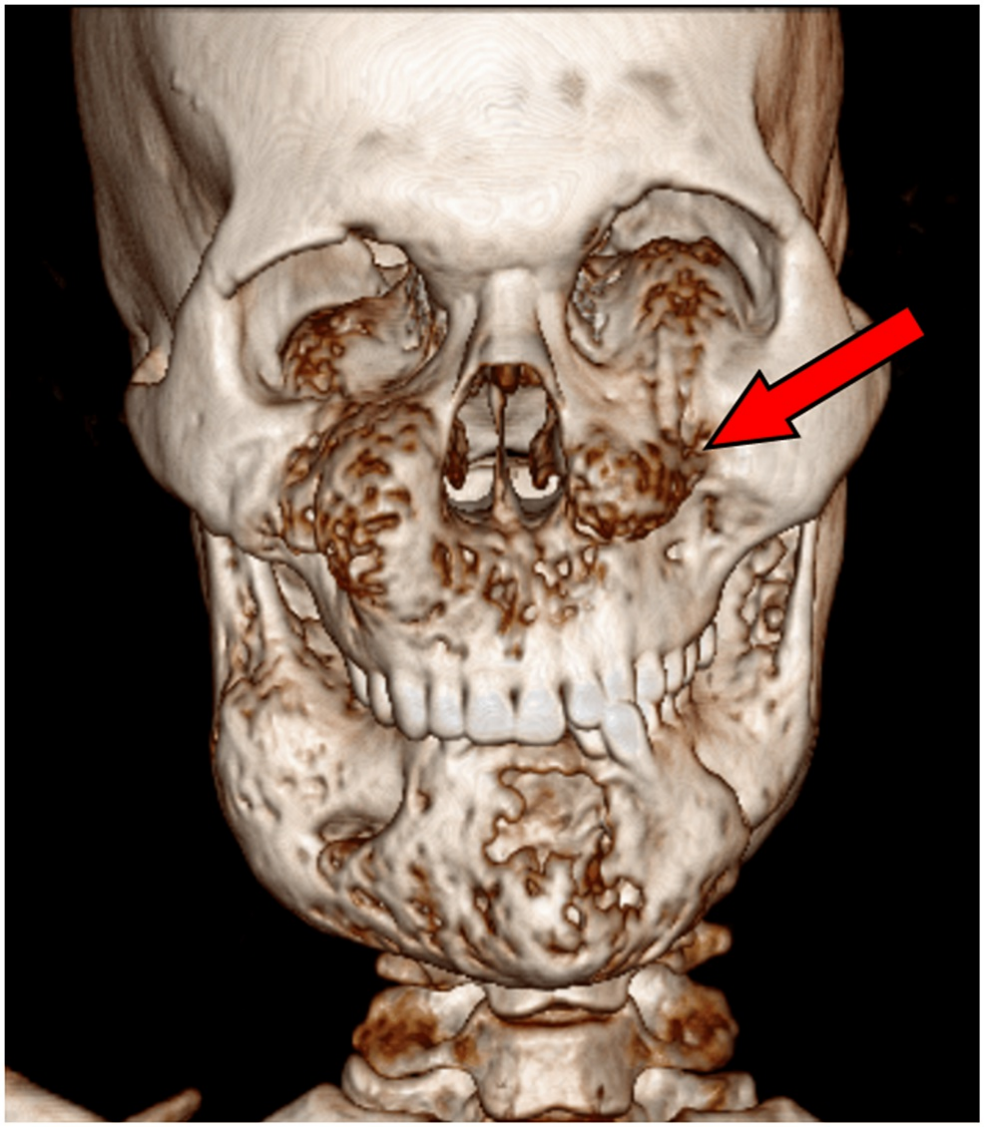
A 3D computed tomography image of a skull This figure shows the expansion of the bony calvarium with an intact cortex and loss of trabeculae, giving a homogeneous ground glass appearance noted in the mandible and maxilla, involving the bilateral floor of orbits, bilateral pterygoid plates, and maxillary sinuses, with partial obscuration of sinuses

Upon inspection, a computed tomography (CT) scan utilizing 3D reconstruction demonstrated a uniform ground-glass look in the mandible and maxilla. This entails a partial obstruction of the sinuses, affecting both the orbital floors, bilateral pterygoid plates, and the maxillary sinuses (Figure 2).

**Figure 3:**
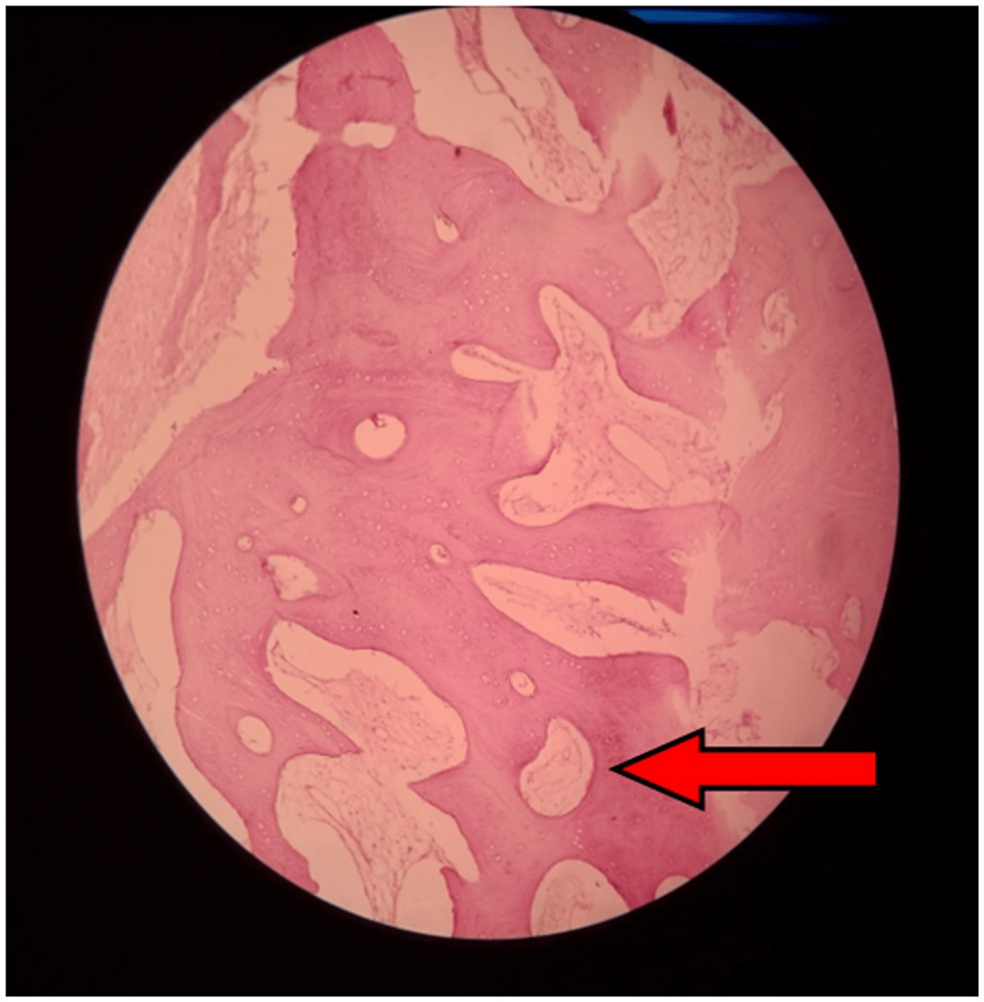
Under low-power view, an H&E stained section shows the presence of multinucleated osteoclast-like giant cells near bone and within soft fibrous stroma, suggestive of cherubism

There was a loss of trabeculae and an intact cortex along with this growth of the bony calvarium. The patient was scheduled for surgery, which involved shaving a portion of bone from both malar areas via an intra-oral maxillary vestibular incision. After that, the incised specimen was sent for histopathological examination. The diagnosis of cherubism was finalized by histopathological analysis (Figure 3).

**Figure 4:**
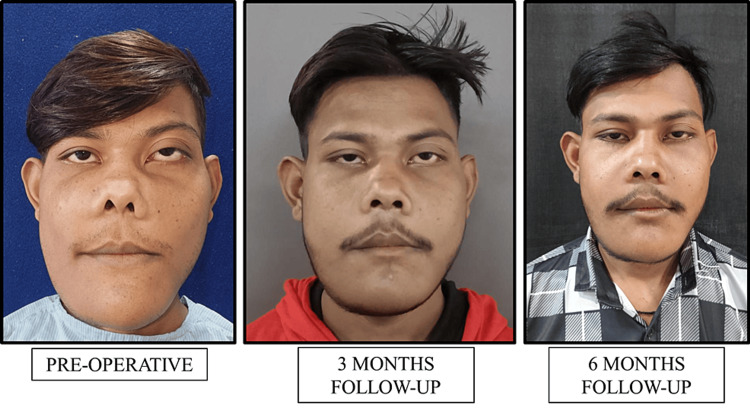
Frontal view of the patient pre-operatively, three-month follow-up, and six-month follow-up, respectively

Following surgery, the patient was prescribed imatinib 200 mg once daily, as advised by the physician. The following figure represents the patient's frontal view pre-operatively, at a three-month follow-up, and at a six-month follow-up, respectively (Figure 4).

## Discussion

Cherubism is an uncommon, benign, hereditary condition characterized by fibro-osseous lesions in the jaws, typically affecting more than one quadrant. This condition becomes stable after the growth period, often resulting in facial disfigurement and malocclusion. Histologically, it exhibits characteristics akin to a giant cell lesion and symmetrically impacts the jaws in children, resulting in a cherubic appearance. Jones introduced the term "cherubism" to delineate the clinical presentation of individuals with this condition. It stands as one of the less common genetically induced bone-resorbing lesions in humans, with a greater prevalence in males and affecting 50-70% of females. The earliest description was given by Jones in 1933 as a familial condition characterized by multiple cyst-like areas affecting the jaw bones, cherubism is believed to result from a mutation in an autosomally inherited gene essential for jaw development. Spontaneous remission of jaw lesions commonly occurs during puberty, although the precise mechanism remains elusive. One hypothesis suggests that the increased synthesis of sex steroids during puberty may counteract the genetic anomaly, thereby mitigating localized osteoclast proliferation in cherubism. The gene linked to cherubism is situated on chromosome 4p16.3, wherein mutations in the SH3BP2 gene are detected in roughly 80% of patients. These mutations lead to the formation of a hyperactive protein involved in cellular signaling, particularly affecting bone remodeling and immune system modulation. This increased activity disrupts normal signaling pathways, causing inflammation in the jawbones and an increase in the production of osteoclasts, leading to the degradation of bone tissue and the formation of cyst-like growths typical of cherubism. Ongoing research aims to delineate the precise role of the SH3BP2 protein in cellular processes and its implications for cherubism and related conditions. To this day, researchers have discovered a sum of 11 mutations in the SH3BP2 gene among individuals diagnosed with cherubism.

Seward and Hankey proposed a grading system of severity and involvement of bones in cherubism, which is outlined in Table 2 [7].

**Table 2:** Seward and Hankey grading system for cherubism

Grading	Clinical features
Grade I	Involvement of bilateral mandibular molar regions and ascending rami, mandible body, or mentis
Grade II	Involvement of bilateral maxillary tuberosities (in addition to grade 1 lesions) and diffuse mandibular involvement
Grade III	Massive involvement of the entire maxilla and mandible, except the condyles
Grade IV	Involvement of both jaws, including the condyles

Cervical lymphadenopathy has been reported in the initial stages of cherubism, mostly it is seen associated with aggressive type [8,9]. From a histological standpoint, examples such as reparative granuloma or brown tumors induced by hyperparathyroidism display multinucleated giant-cell granulomas enmeshed within densely proliferative fibrous connective tissue [10-12]. This tissue contains fibroblasts and multinucleated giant cells resembling osteoclasts, as illustrated in Figure 3. Numerous case studies [13] have indicated that standard blood counts and bone biomarkers, including serum calcium and phosphate concentrations, thyroxine (T4), triiodothyronine (T3), luteinizing hormone (LH), thyroid-stimulating hormone (TSH), follicle-stimulating hormone (FSH), parathyroid hormone (PTH), parathyroid hormone-related protein (PTHrP), calcitonin, and osteocalcin, typically remain within normal ranges. Additionally, there have been reports of a modest elevation in urine deoxypyridinoline levels and an increase in bone-specific alkaline phosphatase [14]. These findings underscore the complexity of the pathophysiological mechanisms involved in cherubism and related conditions, necessitating comprehensive diagnostic evaluations and monitoring protocols for affected individuals.

## Conclusions

In terms of diagnosis, evaluation of risks and co-morbidity, and long-term medical surveillance, cherubism frequently goes unnoticed in Endocrine or Rheumatology Departments, despite the fact that it is usually benign and regressive. The illness presents several difficulties, especially in treating orthodontic issues and dental complications thereafter. While cherubism usually resolves on its own, in extremely aggressive cases that affect vital tissues including tooth eruption pathways, orbital areas, nasal passageways, or cases that cause glossoptosis, surgery becomes necessary. Conservative curettage is usually the recommended surgical procedure in these severe situations due to its less intrusive nature. However, because there is a chance that the affected areas will sustain irreversible harm, its use is controversial. A multidisciplinary approach comprising dental professionals, orthodontists, and, when necessary, surgeons is required for comprehensive care of patients with cherubism in order to address both the functional and cosmetic components of the problem. To properly manage any emerging difficulties and track the course of the condition, long-term follow-up is essential. Improved patient outcomes and comprehensive cherubism management can be achieved by increased awareness and cooperation throughout medical specialties.
